# Adenocarcinoma in bladder diverticulum, metastatic from gastric cancer

**DOI:** 10.1186/1477-7819-3-55

**Published:** 2005-08-24

**Authors:** Nobuhisa Matsuhashi, Kazuya Yamaguchi, Taiso Tamura, Kuniyasu Shimokawa, Yasuyuki Sugiyama, Yosuke Adachi

**Affiliations:** 1 Department of Surgical Oncology, Gifu University Hospital, 1-1 Yanagido, Gifu City 501-1193, Japan; 2 Department of Pathology, Gifu University Hospital, 1-1 Yanagido, Gifu City 501-1193, Japan; 3 Department of Emergency & Disaster Medicine, Gifu University Hospital, 1-1 Yanagido, Gifu City 501-1193, Japan

## Abstract

**Background:**

Metastasis to the urinary bladder from gastric cancer is rare. Metastasis to a diverticulum of the bladder from gastric cancer is extremely rare. We report a case of isolated bladder metastasis from gastric cancer and invasion localized to the muscularis propria of the primary site (stomach).

**Case presentation:**

A 90-year-old female presented with nausea and vomiting that was diagnosed as gastric cancer, the patient also had intermittent hematuria. Pelvic computed tomography identified an abnormally thickened area in the bladder wall that was diagnosed as a diverticulum of the bladder. A biopsy of the bladder wall revealed well differentiated tubular adenocarcinoma metastatic from gastric carcinoma.

**Conclusion:**

Almost all cases of bladder metastasis from gastric cancer had peritoneal dissemination. This particular presentation of bladder metastasis from gastric cancer, to the best of our knowledge, has not been previously reported.

## Background

Metastasis to a diverticulum of the bladder from gastric cancer is extremely rare [1]. Gastric cancer has a tendency to metastasize widely, most commonly to the liver, lung, lymph nodes, bone and peritoneum [2]. The bladder may be involved in the late stages from metastasis and is usually associated with metastasis to other organs [3], but isolated bladder metastasis and invasion localized in the muscularis propria of the primary site (stomach) is extremely rare.

## Case presentation

**Figure 1:**
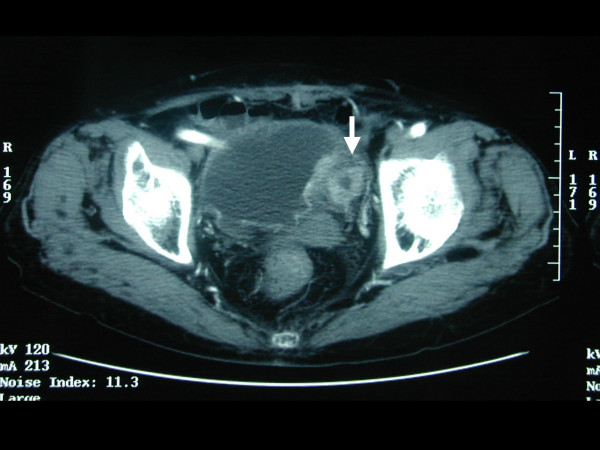
Pelvic computed tomography revealed an abnormally thickened diverticulum of the bladder.

A-90-year-old female presented in November 2003 with a history of nausea, vomiting and dysphagia, with hematuria. On admission her abdomen was slightly distended, tympanic, and slightly tender in the upper abdominal regions, with normal bowel sounds and no palpable mass. Blood tests revealed a white blood cell count of 6,100/mm^3^, c reactive protein (CRP) 6.01 mg/dl, carcinoma antigen (CA) 19-9 50.6 mg/dl and carcinoembryonic antigen (CEA) of 2.9 mg/dl. Pelvic computed tomography (CT) identified an abnormal thickness of the bladder wall with enhance effect in a diverticulum and it's origin from bladder (Figure. 1). Another mass was seen in the antral portion of the stomach; however, pancreas and biliary tract were normal on computed tomography. There was not other lesion detected in other abdominal and pelvic organs.

A gastroscopy was carried out which revealed type 3 pyloric stenosis. A biopsy of the stomach was taken which revealed well-differentiated tubular adenocarcinoma. Cystoscopy was performed which showed a lesion in the bladder diverticulum, a biopsy of the bladder wall revealed well-differentiated tubular adenocarcinoma metastasis from gastric carcinoma. At laparotomy, the pylorus segment of the stomach was viable with signs of edema, but no serosal invasion was identified. There were no sign of peritoneal dissemination in the intra-abdominal cavity. Peritoneal washings were negative for malignant cells. A palliative distal gastrectomy with gastrojejunostomy was performed to relieve pyloric obstruction.

**Figure 2:**
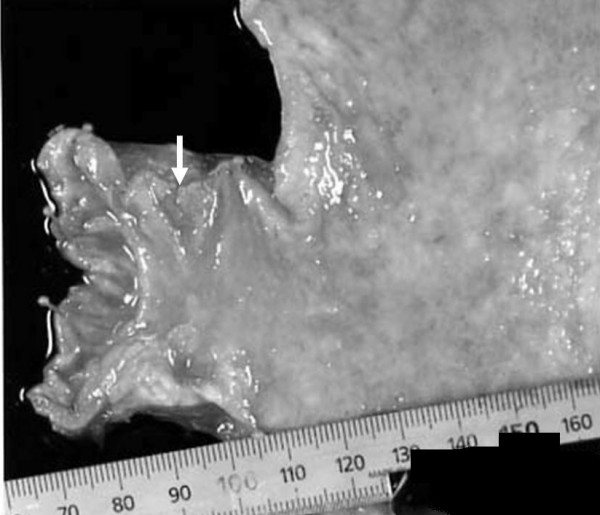
Macroscopic specimens identified a type-3 tumor at the pylorus of the stomach.

**Figure 3:**
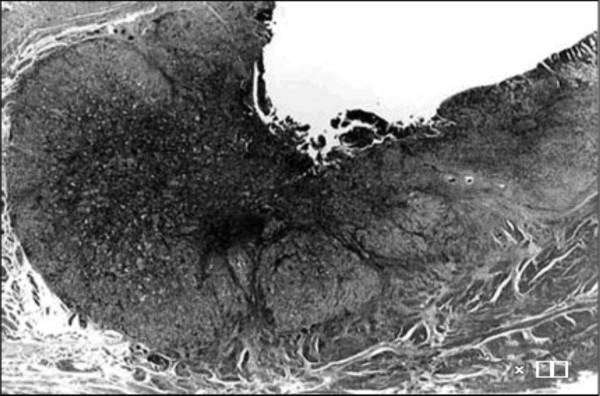
Microscopic specimens demonstrated localization in the muscularis propria on the invasion index (T2) (Hematoxylin and Eosin ×10).

**Figure 4:**
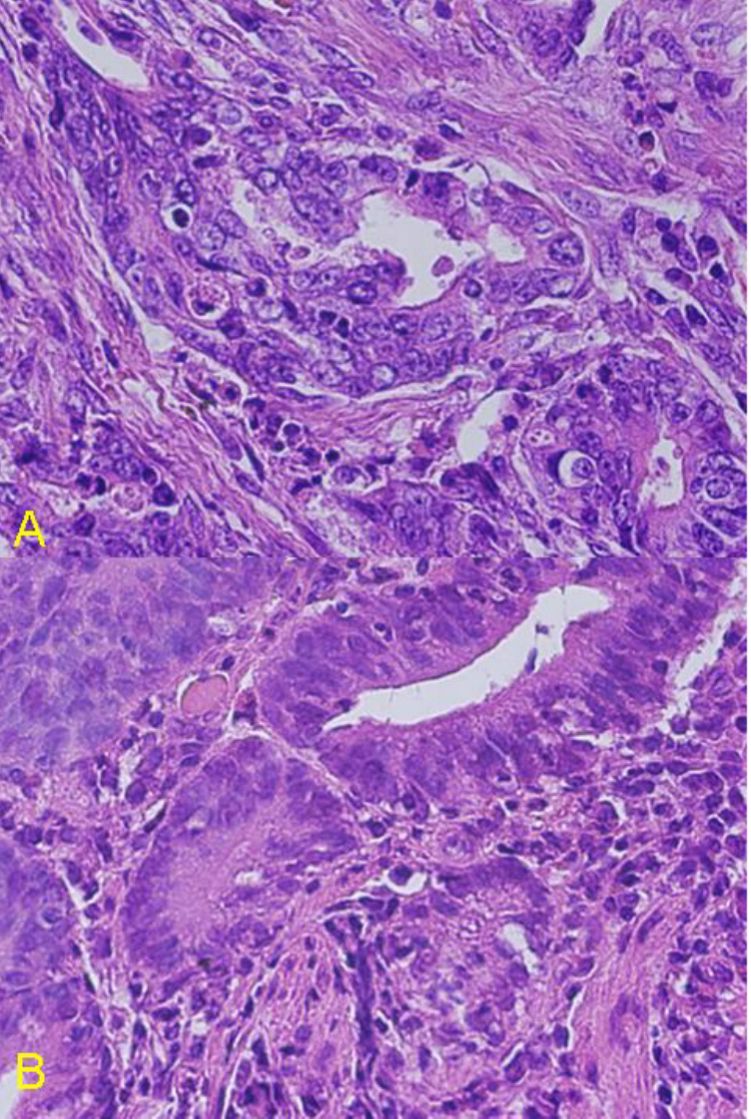
Photomicrograph of a) stomach showing well differentiated tubular adenocarcinoma (left). (Hematoxylin and Eosin ×400) and b) bladder biopsy specimen showing well differentiated tubular adenocarcinoma (right) (Hematoxylin and Eosin ×400).

However, cystectomy or diverticulectomy was not performed due to age of the patient and technical difficulties due to previous two surgeries performed for abnormal position of uterus and volvulus of intestine. The size of the macroscopic specimen was 3.0 × 2.5 cm (Figure. 2). Histology revealed well differentiated tubular adenocarcinoma invading to the muscularis propria (MP), 3type, Infiltrative growth pattern (inf) β, int, ly_3_, v_0 _(Figure. 3, 4a), Similar to that of bladder tumor (Figure 4b). The patient recovered with no further symptoms, and was discharged on the 19th postoperative day. However, patient later developed pyelonephritis, bilateral hydronephrosis, disseminated intravascular coagulation (DIC) and died three months after the surgery.

## Discussion

Metastases to urinary bladder are rare, accounting for less than 2% of all bladder tumors, these are mostly found in advanced stages with peritoneal dissemination [2]. Information pertaining to bladder metastases is derived largely from autopsy studies, and known primary sites of origin in descending frequency are gastric cancer, malignant melanoma, breast and lung [2]. Potential mechanisms contributing to the appearance of secondary bladder tumors from adjacent organs are implantation of exfoliated cells from the bladder periphery or renal pelvis, and lymphogenous, hematogenous, or peritoneal dissemination from a distant primary source [3].

The relative infrequency of primary adenocarcinoma of the bladder causes the dilemma whether bladder adenocarcinoma represents a primary or secondary process [1]. Mostofi *et al*, have proposed several guidelines for such differentiation [2]. If the adjacent mucosa contains polypoid formation, Brunn's nests, or glandular or mucous metaplasia, a primary bladder lesion is likely. An additional feature favoring a bladder origin is the coexistence of transitional and squamous carcinoma. Mostofi *et al*, also stipulated that secondary bladder tumors rarely provoke urinary symptoms before the primary site is detected. In this case, histology indicated that neoplastic columnar cells formed small solid nests and /or small-sized glandular structures. In conclusion, it appears that the stomach was the preponderant site of the origin

More than 95% of the bladder tumors are transitional cell carcinoma and less than 1% is adenocarcinoma [4]. Almost all bladder adenocarcinoma originate from trigone of the bladder. Gastric cancer metastatic to the bladder may behave differently in the two sexes. Among 10 autopsied male patients with gastric cancer, Hermann found bladder metastases in only one; however, among 12 cases of Krukenberg's tumors (ovarian cancers arising from gastrointestinal origin), there were 6 cases with metastases to the bladder, uterus, and Fallopian tubes [3]. It was hypothesized that the ovary might somehow direct metastases to the pelvic organs, since bladder metastases are very rare in the absence of Krukenberg's tumor. Patients of metastatic linitis plastica described by Mizutani *et al*, [4], and Leddy *et al*, [5] were both females with metastatic tumors in at least one ovary as well as the bladder. Since the chief complaint of patients with bladder metastasis is intermittent hematuria, bladder metastases from gastric cancers have been reported mainly by urology surgeons, and thus cancer invasion of the stomach in almost all of the existing case reports was not analyzed [4-7]. Our results indicate that the finding of an abnormally thickened diverticulum of the bladder may provide prognostic value in computed tomography, and additionally in localized gastric cancer lesions with invasion limited to the muscularis propria too might metastasize by lymphogenous spread. To our knowledge this is the first reported case of isolated metastasis to a urinary bladder diverticulum.

## Conclusion

Isolated metastasis to urinary bladder are rare, metastasis to a urinary bladder diverticulum is still rarer.

## Competing interests

The author(s) declare that they have no competing interests.

## Authors' contributions

**NM**, **KY**, **TT**, **YS **and **YA **took part in the operation, performed the literature search and drafted the manuscript for submission.

**KS **performed histological examination and provided photomicrographs.

All authors read and approved the final manuscript.
